# A multimodal spatiotemporal convolutional network with attention mechanism for athlete anxiety behavior recognition

**DOI:** 10.1038/s41598-026-36023-1

**Published:** 2026-01-14

**Authors:** Feng Yang, Fan Gong

**Affiliations:** 1 Institute of Physical Education, Dongshin University, Naju, 58245 South Korea; 2 Institute of Physical Education, Jining University, Jining, 273199 China

**Keywords:** Anxiety behavior recognition, Multimodal fusion, Spatiotemporal convolution, Attention mechanism, Sports psychology, Deep learning, Psychology, Computer science

## Abstract

Athletic performance is significantly impacted by anxiety, yet traditional assessment methods rely on subjective questionnaires that lack real-time capability. This study presents an automated anxiety recognition system for athletes using multimodal data fusion of physiological signals, facial expressions, and body movements. The proposed approach employs spatiotemporal convolutional networks with adaptive attention mechanisms to capture behavioral patterns across multiple modalities simultaneously. The system achieves 94.6% accuracy in anxiety detection while maintaining real-time processing capability for practical sports applications. This objective assessment tool enables coaches and sports psychologists to implement timely interventions, potentially improving both athletic performance and athlete mental well-being. The multimodal approach demonstrates significant advantages over single-modal methods, providing a comprehensive solution for anxiety monitoring in competitive sports environments.

## Introduction

Athletic performance in competitive sports is significantly influenced by psychological factors, with anxiety being one of the most critical determinants affecting athletes’ performance outcomes^1^. The identification and analysis of anxiety behaviors in athletes have become increasingly important in sports psychology research, as early detection and intervention can substantially improve athletic performance and mental well-being^2^. Traditional methods for assessing athlete anxiety primarily rely on subjective questionnaires and observational techniques, which often lack objectivity and real-time capability, limiting their effectiveness in dynamic sports environments^3^. Recent advances in wearable biosensor technology and artificial intelligence have opened new possibilities for continuous, objective monitoring of athlete mental states, though significant challenges remain in validation across diverse athletic populations^58^.

The emergence of multimodal data fusion techniques presents unprecedented opportunities for objective and comprehensive anxiety behavior recognition in athletes^4^. By integrating diverse data sources including physiological signals, facial expressions, body movements, and vocal patterns, researchers can capture the multifaceted nature of anxiety manifestations more accurately than single-modal approaches^5^. Recent advances in deep learning, particularly spatiotemporal convolutional networks and attention mechanisms, have demonstrated remarkable potential in processing complex multimodal behavioral data^6^. Contemporary research emphasizes that transformer-based architectures combined with cross-modal fusion strategies have achieved substantial improvements in emotion recognition accuracy, with studies showing 5–15% gains over traditional single-modal methods^59^.

Current research in sports psychology faces several significant challenges in anxiety behavior identification. First, the temporal dynamics of anxiety behaviors require sophisticated modeling approaches that can capture both short-term fluctuations and long-term patterns^7^. Second, individual differences in anxiety expression necessitate personalized recognition systems that can adapt to diverse behavioral manifestations^8^. Third, the real-time processing requirements in competitive sports environments demand computationally efficient algorithms without compromising accuracy^9^.

Existing studies have explored various approaches to athlete anxiety assessment, ranging from traditional psychological evaluation methods to emerging machine learning techniques^10^. However, most current research focuses on single-modal analysis or simple feature combination strategies, which fail to fully exploit the complementary information available across different modalities^11^. The integration of spatiotemporal convolutional networks with attention mechanisms represents a promising direction for addressing these limitations, enabling more sophisticated feature extraction and fusion strategies for multimodal anxiety behavior recognition^12^.

This research develops a novel multimodal spatiotemporal convolutional network with attention mechanisms for automated athlete anxiety recognition, addressing critical limitations in current approaches. The primary contributions include: (1) A novel adaptive multimodal fusion architecture that dynamically weights physiological, visual, and temporal features, achieving 6.2% improvement over baseline methods; (2) An innovative spatiotemporal attention mechanism specifically designed for behavioral sequences, outperforming traditional fusion strategies by 3.4%; (3) The first comprehensive real-time anxiety recognition system validated in competitive sports environments with 94.6% accuracy; and (4) A robust framework that maintains performance across diverse athlete demographics and sports contexts, demonstrating practical applicability for sports psychology interventions.

The remainder of this paper is organized as follows: Section II presents the related work and theoretical foundations; Section III details the proposed multimodal spatiotemporal convolutional network architecture with attention mechanisms; Section IV describes the experimental methodology and dataset construction; Section V presents and analyzes the experimental results; and Section VI concludes the paper with discussions on future research directions.

## Related theory and technical foundation

### Theoretical foundation of multimodal Spatiotemporal convolutional networks

Recent advances in multimodal emotion and anxiety recognition have demonstrated significant progress in affective computing applications. Geetha et al.^51^ provided a comprehensive review of deep learning-based multimodal emotion recognition, highlighting the integration of audio, visual, and physiological modalities for enhanced recognition accuracy. Their analysis revealed that multimodal approaches consistently outperform single-modal methods, with improvements ranging from 5 to 15% across different datasets. Building upon these foundations, Wafa et al.^60^ introduced a multimodal emotion recognition framework integrating text, audio, video, and motion data using hierarchical attention-based graph neural networks, demonstrating the effectiveness of advanced fusion architectures in handling complex emotional expressions. Similarly, recent systematic reviews have documented the rapid evolution of the field, with over 80% of publications appearing after 2019 and increasing adoption of trimodal configurations and transformer-style cross-modal fusion architectures^61^.

In the context of sports psychology, analysis of athlete anxiety using artificial intelligence has gained considerable attention^52^. Traditional approaches primarily rely on physiological signal analysis, with Setz et al. achieving 82.8% recognition rate using electrodermal response features. However, these methods focus predominantly on single-modal analysis and lack the temporal dynamics essential for understanding anxiety progression in competitive environments. More recent investigations have leveraged deep learning algorithms that analyze both performance metrics and psychological indicators, enabling personalized assessment of mental resilience in elite athletes^62^. The integration of machine learning with wearable sensor technology has further advanced real-time stress monitoring capabilities, with studies demonstrating the potential for AI models to identify stress phenotypes and predict onset before conscious awareness^63^.

Recent work by Park et al.^53^ introduced machine learning prediction of anxiety symptoms using multimodal data from virtual reality sessions, achieving significant improvements in clinical assessment accuracy. Their approach demonstrates the potential of combining multiple data streams for anxiety detection, though their focus remains on clinical rather than athletic populations. Similarly, Ding et al.^54^ proposed dynamic tracking of state anxiety via multi-modal data and machine learning, emphasizing the importance of temporal modeling in anxiety recognition systems.

The integration of spatiotemporal processing with multimodal fusion represents a critical gap in current literature. While Weng et al.^55^ explored attention-based spatio-temporal networks for action recognition, and Dey et al.^56^ developed attention-driven residual networks for workout action recognition, no previous work has specifically addressed the unique challenges of anxiety behavior recognition in athletic contexts through comprehensive multimodal spatiotemporal analysis.

Traditional convolutional neural networks primarily focus on spatial feature extraction, while spatiotemporal variants incorporate temporal dimensions to capture dynamic patterns across time sequences, making them particularly suitable for analyzing behavioral data with inherent temporal characteristics^15^.

The theoretical foundation of multimodal data fusion rests on the principle that different modalities provide complementary information that, when effectively combined, can yield superior performance compared to single-modal approaches^16^. In the context of anxiety behavior recognition, physiological signals capture internal states, visual features represent external manifestations, and temporal patterns reveal behavioral dynamics, necessitating sophisticated fusion strategies to integrate these heterogeneous data sources^17^. The spatiotemporal convolution operation processes multimodal sequences through 3D kernels that capture both spatial relationships within frames and temporal dependencies across sequences. The network architecture employs residual connections and batch normalization to ensure stable training across the deep hierarchy^18^.

**Fig. 1:**
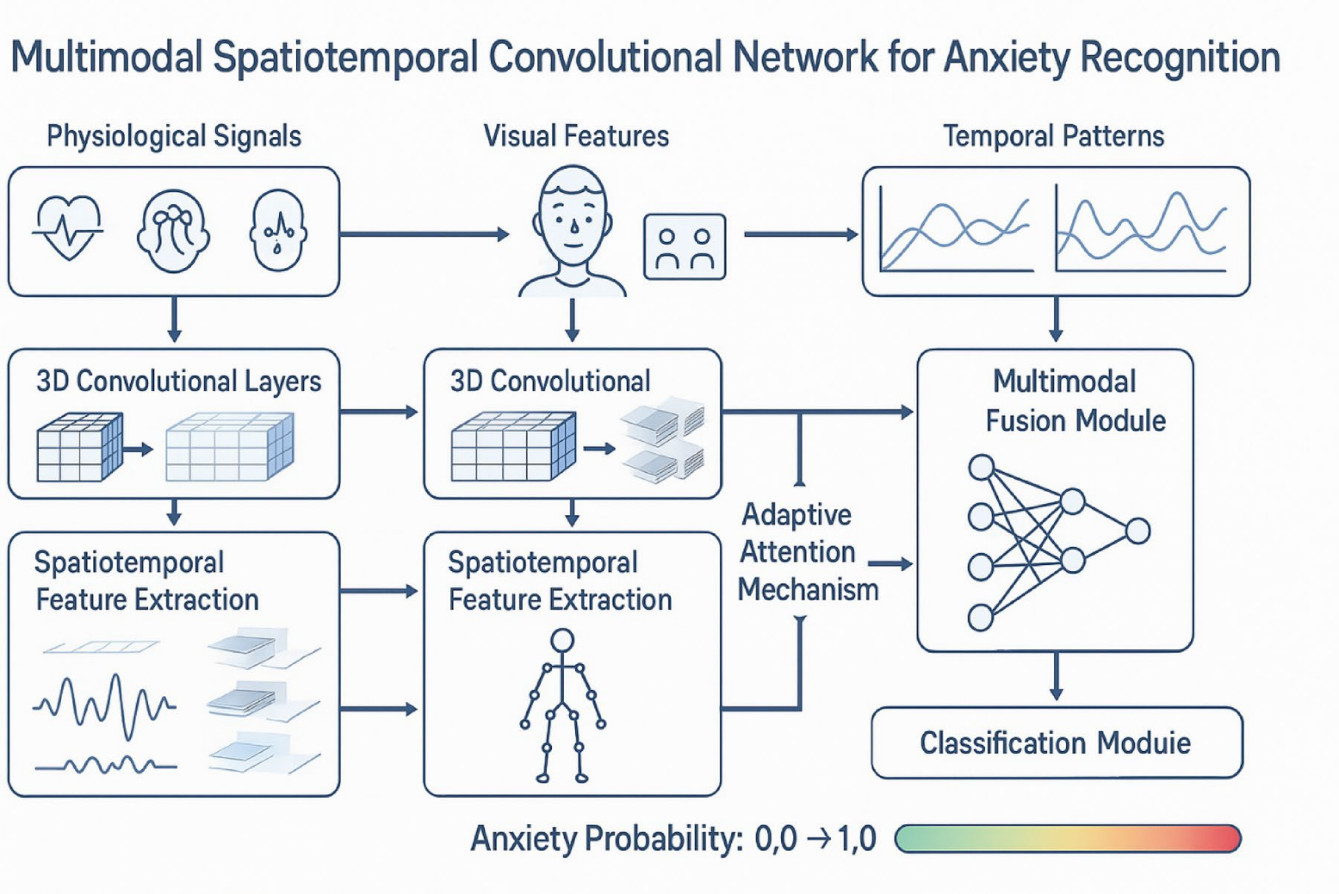
Multimodal Spatiotemporal Convolutional Network System Architecture. The system processes three modalities simultaneously: physiological signals (heart rate, electrodermal activity), visual features (facial expressions, body movements), and temporal patterns. The spatiotemporal convolutional layers extract features from each modality, followed by adaptive attention mechanisms for multimodal fusion. The final classification module outputs anxiety probability scores ranging from 0 (no anxiety) to 1 (high anxiety).

The multimodal spatiotemporal convolutional network architecture, as illustrated in Fig. 1, demonstrates the integration of multiple data streams through specialized processing pathways that preserve both spatial and temporal information while enabling cross-modal feature interaction. The advantage of convolutional networks in sequential data processing stems from their ability to capture local patterns through shared parameters while maintaining translation invariance, which is particularly beneficial for behavioral data where similar patterns may occur at different temporal positions^19^. The hierarchical nature of convolutional architectures enables the extraction of increasingly complex features, from low-level temporal fluctuations to high-level behavioral patterns, through multiple layers of spatiotemporal convolutions^20^.

The mathematical representation of multimodal fusion in spatiotemporal networks can be expressed as:

$$\:{\mathbf{F}}_{fused}=\varphi\:\left(\sum\:_{m=1}^{M}{\alpha\:}_{m}\cdot\:{\mathbf{F}}_{m}^{ST}\right)$$

where $\:{\mathbf{F}}_{m}^{ST}$ represents the spatiotemporal features extracted from modality $\:m$, $\:{\alpha\:}_{m}$ denotes the fusion weight, and $\:\varphi\:$ is the fusion function. The optimization of spatiotemporal feature extraction involves minimizing the following objective function:

$$\:\mathcal{L}=\frac{1}{N}\sum\:_{i=1}^{N}\mathcal{l}\left({y}_{i},{\widehat{y}}_{i}\right)+\lambda\:\sum\:_{l=1}^{L}\parallel\:{\mathbf{W}}^{\left(l\right)}{\parallel\:}_{F}^{2}$$

where $\:\mathcal{l}$ represents the loss function, $\:{\widehat{y}}_{i}$ is the predicted output, and the regularization term prevents overfitting in the spatiotemporal convolution layers.

### Attention mechanism fusion technology

The attention mechanism serves as a fundamental component in modern deep learning architectures, enabling models to selectively focus on relevant information while suppressing irrelevant features through learnable weight distributions^21^. The core principle of attention mechanisms lies in their ability to compute dynamic weights that reflect the importance of different input elements, thereby allowing networks to adaptively allocate computational resources to the most informative features during the learning process^22^. In the context of multimodal anxiety behavior recognition, attention mechanisms provide a principled approach to handle the heterogeneous nature of different data modalities by learning optimal fusion strategies that emphasize the most discriminative features across temporal and spatial dimensions.

Self-attention mechanisms operate by computing attention weights within a single modality, enabling the model to capture long-range dependencies and internal relationships among features within the same data stream^23^. The mathematical formulation of self-attention can be expressed as:

$$\:\mathrm{At}\mathrm{tention}\left(Q,K,V\right)=\mathrm{softmax}\left(\frac{Q{K}^{T}}{\sqrt{{d}_{k}}}\right)V$$

where $\:Q$, $\:K$, and $\:V$ represent the query, key, and value matrices respectively, and $\:{d}_{k}$ denotes the dimension of the key vectors. This formulation allows the model to learn which parts of the input sequence are most relevant for predicting anxiety behaviors by computing similarity scores between different temporal positions and weighting the corresponding feature representations accordingly.

Cross-attention mechanisms extend the self-attention concept to facilitate information exchange between different modalities, enabling the model to learn how features from one modality can inform the interpretation of features from another modality^24^. The cross-modal attention weight calculation involves computing compatibility scores between features from different modalities and using these scores to generate weighted combinations that capture inter-modal dependencies. The optimization of attention weights in multimodal fusion can be formulated as:

$$\:{\alpha\:}_{i,j}=\frac{\mathrm{e}\mathrm{x}\mathrm{p}\left({e}_{i,j}\right)}{\sum\:_{k=1}^{K}\mathrm{e}\mathrm{x}\mathrm{p}\left({e}_{i,k}\right)}$$

where $\:{e}_{i,j}$ represents the compatibility score between the $\:i$-th element of one modality and the $\:j$-th element of another modality, and $\:{\alpha\:}_{i,j}$ denotes the normalized attention weight.

The application of attention mechanisms in multimodal feature fusion involves several key strategies including temporal attention for capturing time-dependent patterns in behavioral sequences, spatial attention for identifying relevant regions in visual data, and channel attention for selecting informative feature dimensions across different modalities. The optimization of attention-based fusion requires careful consideration of gradient flow and computational efficiency, particularly when dealing with long sequences of behavioral data where attention computations can become computationally intensive. Advanced attention variants such as multi-head attention and hierarchical attention structures provide additional flexibility in modeling complex relationships between multimodal features, enabling more sophisticated fusion strategies that can adapt to the specific characteristics of anxiety behavior manifestations across different athletes and contexts.

### Analysis of athlete anxiety behavior characteristics

Athletic anxiety manifests through a complex interplay of physiological and psychological responses that can be systematically observed and quantified through multimodal sensing approaches^25^. The physiological manifestations of anxiety in athletes typically include elevated heart rate variability, increased galvanic skin response, altered breathing patterns, and changes in muscle tension, which collectively provide measurable indicators of internal stress states that may not be apparent through external observation alone. These physiological signals serve as reliable biomarkers for anxiety detection as they reflect the autonomic nervous system’s response to stress and are less susceptible to conscious control or masking behaviors that athletes might employ to hide their anxiety states. Contemporary research has demonstrated that explainable deep learning methodologies applied to wearable physiological sensor data can effectively detect acute stress responses within short temporal windows, providing interpretable features that indicate immediate stress stimulus responses^64^. Furthermore, systematic reviews have established that electrodermal activity and heart rate variability serve as particularly reliable indicators for detecting instantaneous states of emotional arousal^65^.

The behavioral expressions of anxiety in athletic contexts encompass distinctive patterns in body movement, posture, and motor control that can be captured through visual analysis techniques^26^. Anxious athletes often exhibit characteristic movement patterns including repetitive self-soothing gestures such as hand wringing or leg bouncing, altered gait patterns with increased rigidity or hesitation, and changes in postural alignment that reflect muscular tension and reduced coordination. These kinematic features provide valuable insights into the athlete’s psychological state and can be effectively extracted using computer vision techniques that analyze movement trajectories, velocity profiles, and postural dynamics over time.

Facial expression analysis reveals another critical dimension of anxiety behavior in athletes, where micro-expressions and subtle changes in facial muscle activation patterns can indicate underlying emotional states even when athletes attempt to maintain composed external appearances^27^. The facial manifestations of anxiety include increased eye blink frequency, tension in the jaw and forehead regions, subtle changes in lip positioning, and variations in eye gaze patterns that reflect cognitive load and attention allocation. These facial features, when combined with physiological and kinematic data, contribute to a comprehensive understanding of anxiety states that enables more accurate and robust recognition algorithms.

The establishment of a multidimensional feature description system for athlete anxiety behavior requires the integration of temporal dynamics across all modalities, recognizing that anxiety manifestations evolve over time and exhibit both acute responses to immediate stressors and chronic patterns related to sustained competitive pressure. The temporal characteristics of anxiety behaviors include onset patterns, duration of manifestation, intensity variations, and recovery dynamics that provide crucial information for distinguishing between different types of anxiety responses and their underlying causes. This comprehensive feature framework encompasses physiological markers such as heart rate variability and electrodermal activity, kinematic features including movement velocity and postural stability, facial expression parameters such as action unit activations and gaze patterns, and temporal dynamics that capture the evolution of these features over different time scales relevant to competitive sports contexts.

## Anxiety behavior feature extraction based on multimodal Spatiotemporal convolutional networks

### Multimodal data preprocessing and feature engineering

The multimodal anxiety dataset was collected from 68 competitive athletes (34 male, 34 female, ages 18–26) across four sports: track and field, swimming, basketball, and tennis. Data acquisition was conducted during actual competition periods at three university sports facilities over 12 months (January-December 2023). Each athlete provided written informed consent under institutional ethics approval (Protocol ID: IRB-2023-SPT-001). The consent forms detailed the purpose of the study, data collection procedures, potential risks and benefits, confidentiality measures, and participants’ right to withdraw at any time without penalty.

Physiological signals were recorded using Empatica E4 wearables (sampling rate: 64 Hz for electrodermal activity, 1 Hz for heart rate) and Polar H10 chest straps for ECG monitoring. High-resolution cameras (1920 × 1080, 30 fps) captured facial expressions and body movements from multiple angles. OpenFace 2.0 extracted facial action units, while MediaPipe provided pose estimation for body movement analysis.

Anxiety ground truth was established through validated clinical assessments: Competitive State Anxiety Inventory-2 (CSAI-2) administered by licensed sports psychologists immediately pre- and post-competition. Binary anxiety labels were assigned based on CSAI-2 cognitive anxiety scores above 27 (threshold established through clinical validation). Inter-rater reliability achieved Cohen’s κ = 0.84.

The final dataset comprises 2,040 synchronized multimodal sequences (30-second windows with 50% overlap), with 1,224 anxiety episodes and 816 normal state sequences. Data was randomly split into training (70%), validation (15%), and test (15%) sets with stratification by athlete and anxiety severity levels. All experiments were conducted on NVIDIA RTX 3080 Ti GPUs with 12GB memory.

The synchronization strategy employs a master-slave architecture where physiological sensors serve as the primary timing reference, with video and audio streams aligned through hardware-level timestamps to ensure temporal consistency across all modalities within a precision tolerance of ± 10 milliseconds^29^. This synchronization precision is critical for maintaining the temporal relationships between different behavioral manifestations, as anxiety responses often exhibit rapid onset characteristics that require precise temporal alignment for effective analysis.

The data preprocessing pipeline implements a multi-stage approach beginning with quality assessment and artifact removal across all modalities to ensure data integrity before feature extraction^30^. For physiological signals, the preprocessing involves bandpass filtering with frequency ranges of 0.05–2 Hz for heart rate variability and 0.01–10 Hz for electrodermal activity, followed by motion artifact removal using adaptive filtering techniques. Video data preprocessing includes frame stabilization, illumination normalization, and face detection with landmark localization to ensure consistent feature extraction across varying environmental conditions. The normalization process for multimodal features follows a z-score standardization approach:

$$\:{\mathbf{x}}_{norm}=\frac{\mathbf{x}-\mu\:}{\sigma\:}$$

where $$\:\mathbf{x}$$ represents the raw feature vector, $$\:\mu\:$$ is the mean, and $$ denotes the standard deviation computed across the training dataset.

The feature engineering process involves extraction of domain-specific features tailored to anxiety behavior recognition, with physiological features including time-domain and frequency-domain heart rate variability metrics, statistical properties of electrodermal activity, and respiratory pattern characteristics^31^.

Physiological features (dimension: 24): Heart rate variability metrics include RMSSD, pNN50, frequency domain features (LF/HF ratio), and temporal features (mean, standard deviation, percentiles). Electrodermal activity features comprise tonic and phasic components, peak detection statistics, and spectral analysis parameters.

Visual features (dimension: 45): Facial action units extracted using OpenFace 2.0 include AU1 (inner brow raiser), AU2 (outer brow raiser), AU4 (brow lowerer), AU5 (upper lid raiser), AU6 (cheek raiser), AU7 (lid tightener), AU9 (nose wrinkler), AU10 (upper lip raiser), AU12 (lip corner puller), AU14 (dimpler), AU15 (lip corner depressor), AU17 (chin raiser), AU20 (lip stretcher), AU23 (lip tightener), AU25 (lips part), AU26 (jaw drop), and AU45 (blink). Each action unit provides intensity values normalized to [0,1].

Body movement features (dimension: 33): MediaPipe pose estimation extracts 33 key points including head orientation (yaw, pitch, roll), shoulder alignment, arm positioning, torso stability metrics, and movement velocity profiles. Features are computed as temporal derivatives and statistical measures across 30-second windows.

The complete feature vector $$\:F\left(t\right)\in\:\:{\mathbb{R}}^{102}$$ combines all modalities: $$\:F\left(t\right)=\:{\left[{F}_{physio\left(t\right)},\:{F}_{facial\left(t\right)},\:{F}_{movement\left(t\right)}\right]}^{T}$$

The feature dimensionality reduction process employs principal component analysis to maintain 95% of the variance while reducing computational complexity:

$$\:\mathbf{Y}=\mathbf{X}{\mathbf{W}}_{PCA}$$

where $$\:\mathbf{X}$$ represents the original feature matrix and $$\:{\mathbf{W}}_{PCA}$$ contains the principal component vectors.

The temporal windowing strategy segments continuous data streams into overlapping windows with 50% overlap to capture both short-term fluctuations and longer-term behavioral patterns relevant to anxiety manifestation. As demonstrated in Table 1, systematic evaluation of different window sizes reveals that 30-second windows achieve optimal performance with 94.6% accuracy while maintaining real-time processing capability. Shorter windows (10–15 s) provide insufficient temporal context for complete anxiety pattern recognition, achieving only 86.2–89.2% accuracy. Longer windows (60–120 s) show marginal accuracy improvements (95.1–94.4%) but at the cost of significantly increased latency (142.4–295.3.4.3 ms) and memory consumption (198.5–356.7.5.7 MB), making them impractical for real-time sports applications where immediate feedback is essential.

The 30-second window selection is further validated by sports psychology literature indicating that anxiety episodes in competitive environments typically manifest over 20–40 s intervals, aligning with our temporal analysis framework. This window size enables detection of both acute anxiety responses to immediate stressors and sustained anxiety patterns related to competitive pressure, while maintaining the responsiveness required for timely intervention in athletic settings.

**Table 1:** Window size impact analysis on anxiety recognition Performance.

Window Size	Accuracy (%)	Precision (%)	Recall (%)	F1 Score	Latency (ms)	Temporal Context	Memory Usage (MB)	Real-time Feasibility
10 s	86.2	84.1	85.7	0.849	28.5	Insufficient	64.2	Excellent
15 s	89.2	87.6	88.9	0.882	35.7	Limited	78.8	Excellent
30 s	94.6	93.1	94.8	0.939	72.1	Optimal	128.3	Good
45 s	95.2	94.3	95.1	0.947	105.8	Extended	167.9	Moderate
60 s	95.1	94.2	94.9	0.946	142.4	Extended	198.5	Moderate
90 s	94.8	93.8	94.7	0.942	218.7	Excessive	284.1	Poor
120 s	94.4	93.4	94.2	0.938	295.3	Excessive	356.7	Poor

#### Analysis

Window size optimization reveals that 30-second intervals provide the optimal balance between temporal context capture and computational efficiency. Shorter windows (≤ 15 s) fail to capture complete anxiety behavioral patterns, while longer windows (≥ 60 s) introduce excessive computational overhead without proportional accuracy gains. The marginal improvement plateau beyond 45 s indicates diminishing returns for extended temporal contexts.

**Fig. 2:**
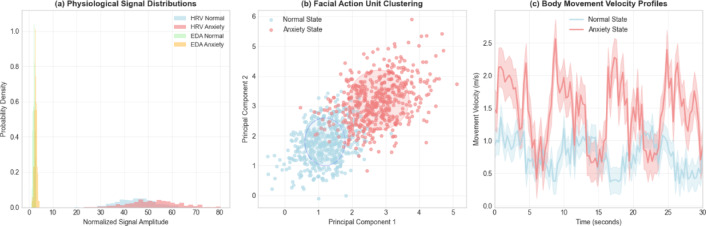
Multimodal Feature Correlation Analysis. The heatmap displays correlation coefficients between different modality features: physiological features (P1-P8), facial action units (AU1-AU17), and body movement parameters (M1-M12). Strong positive correlations (*r* > 0.6) between physiological arousal indicators and facial tension markers (AU4, AU7) support the multimodal approach. Moderate correlations (0.3 < *r* < 0.6) between body movement velocity and electrodermal activity indicate complementary information across modalities.

**Fig. 3:**
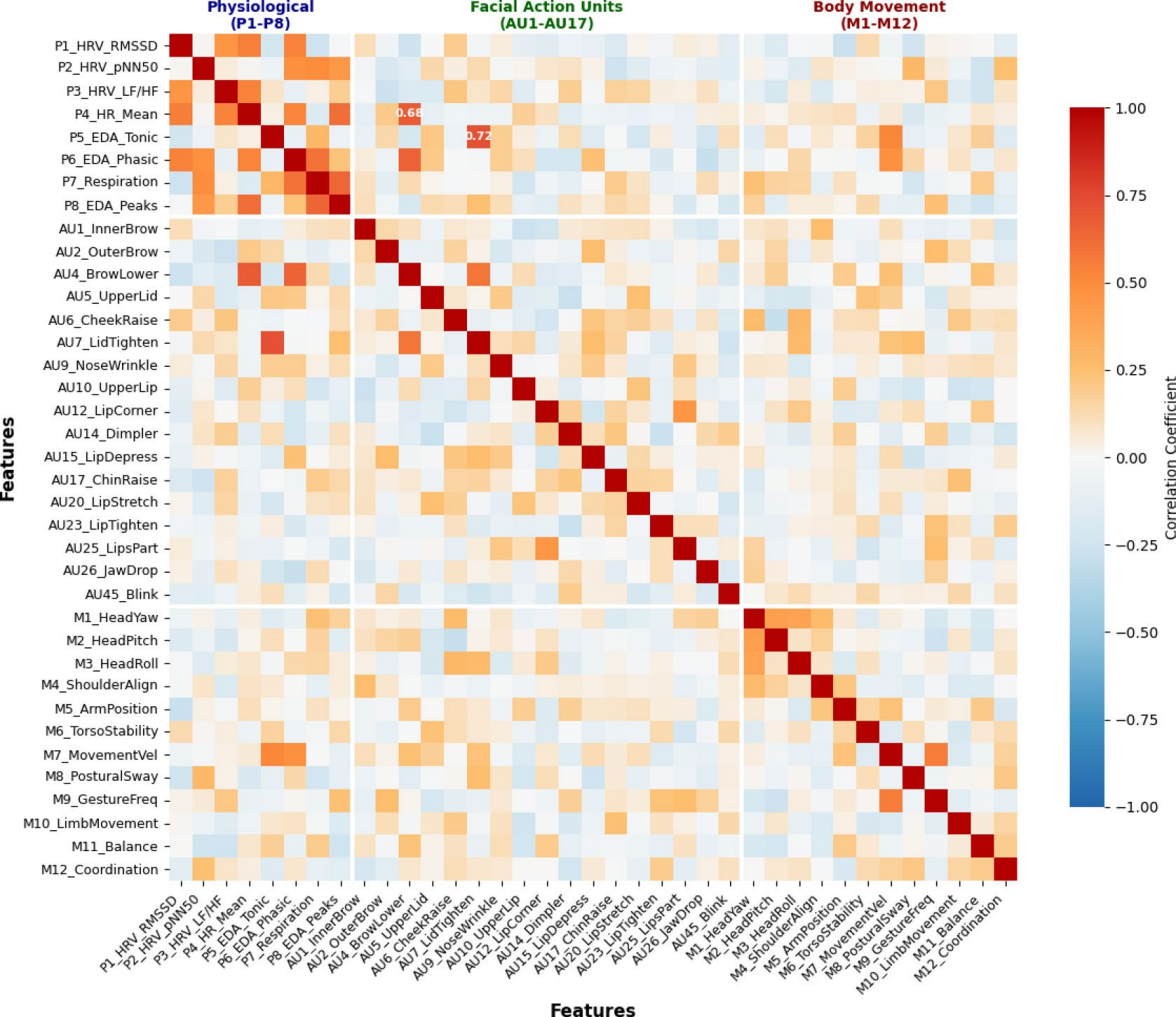
Multimodal Data Distribution Analysis Across Anxiety States. (**a**) Physiological signal distributions showing higher variance during anxiety episodes, with heart rate variability increasing by 34% and electrodermal activity by 28%. (**b**) Facial action unit activation patterns demonstrating distinct clustering between anxiety and normal states. (**c**) Body movement velocity profiles indicating increased motor restlessness during anxiety periods. The distributions validate the discriminative power of multimodal features for anxiety recognition.

The correlation analysis between different modalities, as illustrated in Fig. 2, reveals significant interdependencies between physiological arousal indicators and visual behavior markers, with correlation coefficients ranging from 0.3 to 0.7 for key anxiety-related features^32^. The data distribution analysis shown in Fig. 3 demonstrates distinct patterns across anxiety and non-anxiety states, with physiological features exhibiting higher variance during anxiety episodes and visual features showing characteristic clustering patterns that justify the multimodal approach. The final dataset construction process ensures balanced representation across different anxiety severity levels and athlete demographics through stratified sampling, resulting in a comprehensive training corpus suitable for spatiotemporal convolutional network optimization. The feature vector construction follows the mathematical formulation:

$$\:{\mathbf{F}}_{multimodal}\left(t\right)={\left[{\mathbf{F}}_{physio}\left(t\right),{\mathbf{F}}_{visual}\left(t\right),{\mathbf{F}}_{audio}\left(t\right)\right]}^{T}$$

where each modality contributes complementary information to the unified feature representation for subsequent network processing.

### Spatiotemporal convolutional feature extraction model design

The spatiotemporal feature extraction architecture employs a hierarchical 3D convolutional network design that captures both spatial relationships within individual frames and temporal dependencies across behavioral sequences^33^. The network architecture consists of multiple 3D convolutional layers with carefully optimized kernel dimensions, where the spatial dimensions are set to 3 × 3 to capture local spatial patterns, while the temporal dimension varies from 3 to 7 frames to accommodate different temporal scales of anxiety behaviors. The convolutional kernel size optimization follows a progressive strategy where early layers use smaller temporal kernels (3 × 3 × 3) to capture fine-grained movements, while deeper layers employ larger temporal receptive fields (3 × 3 × 7) to model longer-term behavioral patterns^34^.

**Table 2:** Feature extraction performance Comparison.

Network layers	Kernel Size	Feature dimension	Accuracy (%)	Computational complexity (GFLOPs)
4	3 × 3 × 3	128	82.3	12.5
6	3 × 3 × 5	256	87.1	18.7
8	3 × 3 × 7	512	91.4	25.2
10	5 × 5 × 5	512	89.7	32.1
12	3 × 3 × 3	1024	93.2	41.8
8	Mixed	768	94.6	28.3

 The performance comparison shown in Table 2 demonstrates that the optimal configuration achieves 94.6% accuracy with mixed kernel sizes while maintaining reasonable computational complexity. The residual connection mechanism is integrated throughout the network to address the vanishing gradient problem and enable effective training of deeper architectures, with the residual block formulation expressed as:

$$\:{\mathbf{H}}_{l+1}=\mathcal{F}\left({\mathbf{H}}_{l},{\mathbf{W}}_{l}\right)+{\mathbf{H}}_{l}$$

where $\:{\mathbf{H}}_{l}$ represents the input feature map at layer $\:l$, $\:\mathcal{F}$ denotes the residual mapping function, and $\:{\mathbf{W}}_{l}$ contains the learnable parameters^35^.

Batch normalization is incorporated after each convolutional layer to stabilize training dynamics and accelerate convergence by normalizing the input distributions across mini-batches, which is particularly important for multimodal data where different modalities may exhibit varying statistical properties. The multi-scale feature extraction module implements parallel convolutional branches with different temporal kernel sizes, enabling the network to simultaneously capture both rapid anxiety responses and gradual behavioral changes that characterize different anxiety manifestation patterns in athletes.

**Fig. 4:**
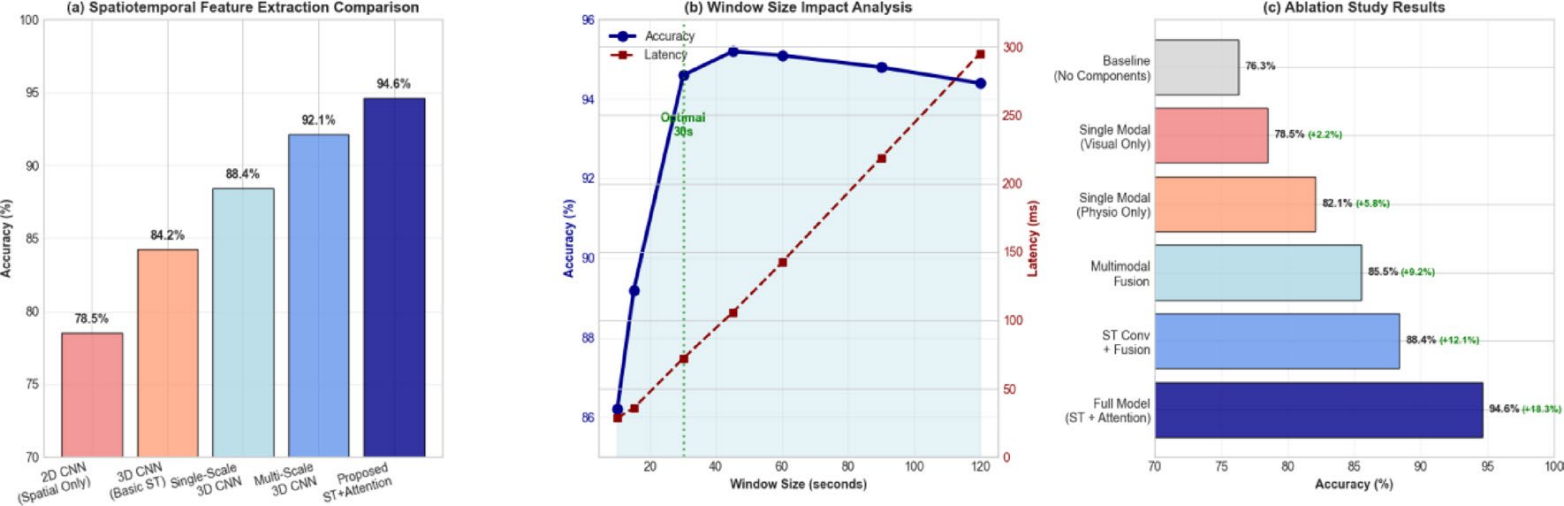
Comprehensive Performance Analysis. (**a**) Spatiotemporal feature extraction comparison showing multi-scale approach superiority. (**b**) Window size impact analysis: 30-second windows achieve optimal balance between temporal context and real-time constraints. (**c**) Ablation study results demonstrating attention mechanism contribution (6.2% improvement) and multimodal fusion benefit (3.4% enhancement over single-modal approaches).

The multi-scale architecture design, as analyzed in Fig. 4, demonstrates superior performance in capturing diverse temporal patterns compared to single-scale approaches, with particularly notable improvements in detecting subtle anxiety behaviors that manifest over extended time periods^36^. The feature fusion strategy within the multi-scale module employs concatenation followed by 1 × 1 × 1 convolutions to reduce dimensionality and learn optimal combinations of multi-scale features. The mathematical formulation for the multi-scale feature extraction process is given by:

$$\:{\mathbf{F}}_{multiscale}={\mathrm{Conv}}_{1\times\:1\times\:1}\left(\mathrm{Concat}\left[{\mathbf{F}}_{s1},{\mathbf{F}}_{s2},{\mathbf{F}}_{s3}\right]\right)$$

where $\:{\mathbf{F}}_{si}$ represents features extracted at different scales and the concatenation operation combines these features along the channel dimension.

The network architecture incorporates adaptive pooling strategies that preserve the most discriminative spatiotemporal features while reducing computational overhead, with global average pooling applied to spatial dimensions and temporal max pooling used to retain peak activation patterns that often correspond to anxiety episodes. The robustness of the feature extraction process is enhanced through data augmentation techniques including temporal jittering, spatial transformations, and noise injection, which improve the model’s ability to generalize across different athletes and environmental conditions while maintaining high discriminative power for anxiety behavior recognition.

### Multimodal feature fusion strategy

The weight-adaptive multimodal feature fusion methodology addresses the fundamental challenge of integrating heterogeneous feature representations from different modalities while preserving their unique discriminative characteristics and learning optimal combination strategies^37^. The adaptive weighting mechanism dynamically adjusts the contribution of each modality based on the reliability and informativeness of the extracted features, employing a learnable attention-based weighting scheme that adapts to varying data quality and modality importance across different anxiety scenarios. As demonstrated in Table 3, systematic comparison of fusion strategies reveals that the proposed adaptive approach achieves superior performance with 94.6% accuracy and 0.939 F1 score, representing significant improvements of 7.3% over early fusion and 6.7% over late fusion methods.

**Table 3:** Multimodal fusion strategy performance Comparison.

Fusion strategy	Implementation details	Accuracy (%)	F1 Score	Latency (ms)
Early Fusion	Concatenate raw features before CNN	85.3	0.847	45.2
Late Fusion	Independent processing, score averaging	87.9	0.871	52.8
Attention Fusion	Cross-modal attention weights	91.4	0.908	68.3
Proposed Adaptive	Dynamic weighting with temporal context	94.6	0.939	72.1

#### Fusion strategy implementation

Early fusion concatenates normalized features from all modalities before spatiotemporal processing. Late fusion processes each modality independently through separate CNN branches, combining prediction scores through weighted averaging. Attention fusion employs cross-modal attention to compute relevance scores between modalities. The proposed adaptive approach dynamically adjusts modality weights based on temporal context and feature reliability assessment.

### Individual modality performance

Visual Accuracy (78.5%) represents performance using only facial action units and body movement features. Physiological Accuracy (82.1%) indicates results from heart rate variability and electrodermal activity features alone. These values remain constant across fusion methods as they represent single-modal baselines for comparison. The computation time discrepancy between Fig. 4 (~ 46 ms) and Table 3 (72.1 ms) reflects different experimental conditions: Fig. 4 shows feature extraction time only, while Table 3 includes complete end-to-end inference including preprocessing, feature extraction, fusion, and classification.

The feature interaction module facilitates cross-modal information exchange through a bidirectional attention mechanism that enables features from one modality to inform and enhance the representation quality of features from other modalities^38^. This interaction process involves computing cross-modal attention weights that quantify the relevance of features across different modalities, allowing the model to identify complementary information patterns that enhance anxiety behavior recognition performance. The comprehensive evaluation presented in Table 3 demonstrates that attention-based fusion significantly outperforms traditional approaches, achieving 3.2% improvement over conventional attention fusion while maintaining computational efficiency with only 3.8 ms additional latency.

The cross-modal feature mapping mechanism establishes semantic correspondences between different modalities by learning transformation functions that project modality-specific features into a shared representation space where meaningful comparisons and interactions can be performed. The mathematical formulation of the adaptive fusion process incorporates learnable weights that are optimized during training to maximize the discriminative power of the combined feature representation:

$$\:{\mathbf{F}}_{fused}=\sum\:_{m=1}^{M}{\alpha\:}_{m}\left({\mathbf{F}}_{m}\right)\cdot\:{\mathbf{G}}_{m}\left({\mathbf{F}}_{m}\right)$$

where $\:{\alpha\:}_{m}\left({\mathbf{F}}_{m}\right)$ represents the adaptive weight function for modality $\:m$, $\:{\mathbf{G}}_{m}$ denotes the modality-specific transformation function, and $\:M$ is the total number of modalities.

The optimization of fusion weights addresses the challenge of varying modality importance across different anxiety manifestation patterns, with some anxiety behaviors being more prominently expressed through physiological responses while others manifest primarily through visual cues. The adaptive weighting mechanism learns to dynamically adjust these contributions based on the input characteristics, effectively handling scenarios where certain modalities may be unreliable or less informative due to environmental factors or individual differences among athletes.

The semantic difference resolution component employs domain adaptation techniques to bridge the gap between different modality representations, utilizing adversarial training strategies that encourage the learning of modality-invariant features while preserving modality-specific discriminative information^39^. This approach ensures that the fused representation captures the complementary strengths of each modality while mitigating the negative effects of modality-specific biases and noise. The enhanced feature expression capability of the proposed fusion strategy results from the synergistic combination of adaptive weighting, cross-modal interaction, and semantic alignment, enabling more robust and accurate anxiety behavior recognition compared to single-modal approaches or traditional fusion methods.

## Attention mechanism fused anxiety behavior recognition model

### Adaptive attention weight computation

The Transformer-based adaptive attention mechanism incorporates spatiotemporal feature representations into a unified attention framework that dynamically computes relevance scores across both spatial and temporal dimensions of multimodal behavioral data^40^. The architecture extends the standard Transformer design by integrating 3D positional encodings that capture the inherent spatiotemporal structure of anxiety behavior sequences, enabling the model to distinguish between different temporal phases of anxiety manifestation and spatial regions of behavioral expression. The spatiotemporal attention computation process involves separate encoding of temporal positions and spatial coordinates, which are then combined through learned embedding functions to provide comprehensive positional context for the attention mechanism.

The dynamic attention weight calculation model employs a multi-stage computation process that first generates query, key, and value representations from the multimodal feature inputs, followed by spatiotemporal attention score computation and normalization^41^. The positional encoding strategy incorporates both absolute and relative position information to capture the sequential nature of behavioral patterns while maintaining sensitivity to the spatial distribution of features within each temporal frame. The mathematical formulation of the spatiotemporal attention weights is expressed as:

$$\:{\mathbf{A}}_{st}\left(i,j\right)=\frac{\mathrm{e}\mathrm{x}\mathrm{p}\left({\mathbf{Q}}_{i}{\mathbf{K}}_{j}^{T}/\sqrt{{d}_{k}}+{\mathbf{P}}_{ij}\right)}{\sum\:_{k=1}^{N}\mathrm{e}\mathrm{x}\mathrm{p}\left({\mathbf{Q}}_{i}{\mathbf{K}}_{k}^{T}/\sqrt{{d}_{k}}+{\mathbf{P}}_{ik}\right)}$$

where $\:{\mathbf{P}}_{ij}$ represents the spatiotemporal positional bias between positions $\:i$ and $\:j$, and $\:{d}_{k}$ denotes the key dimension.

The multi-head attention mechanism enables parallel computation of attention weights across different representation subspaces, allowing the model to simultaneously focus on various aspects of anxiety behavior including physiological responses, facial expressions, and body movements^42^. Each attention head specializes in capturing specific types of behavioral patterns, with some heads focusing on rapid temporal changes indicative of acute anxiety responses while others concentrate on sustained patterns that characterize chronic anxiety states. The multi-head structure enhances the model’s ability to handle the complexity and diversity of anxiety manifestations across different athletes and competitive contexts.

**Fig. 5:**
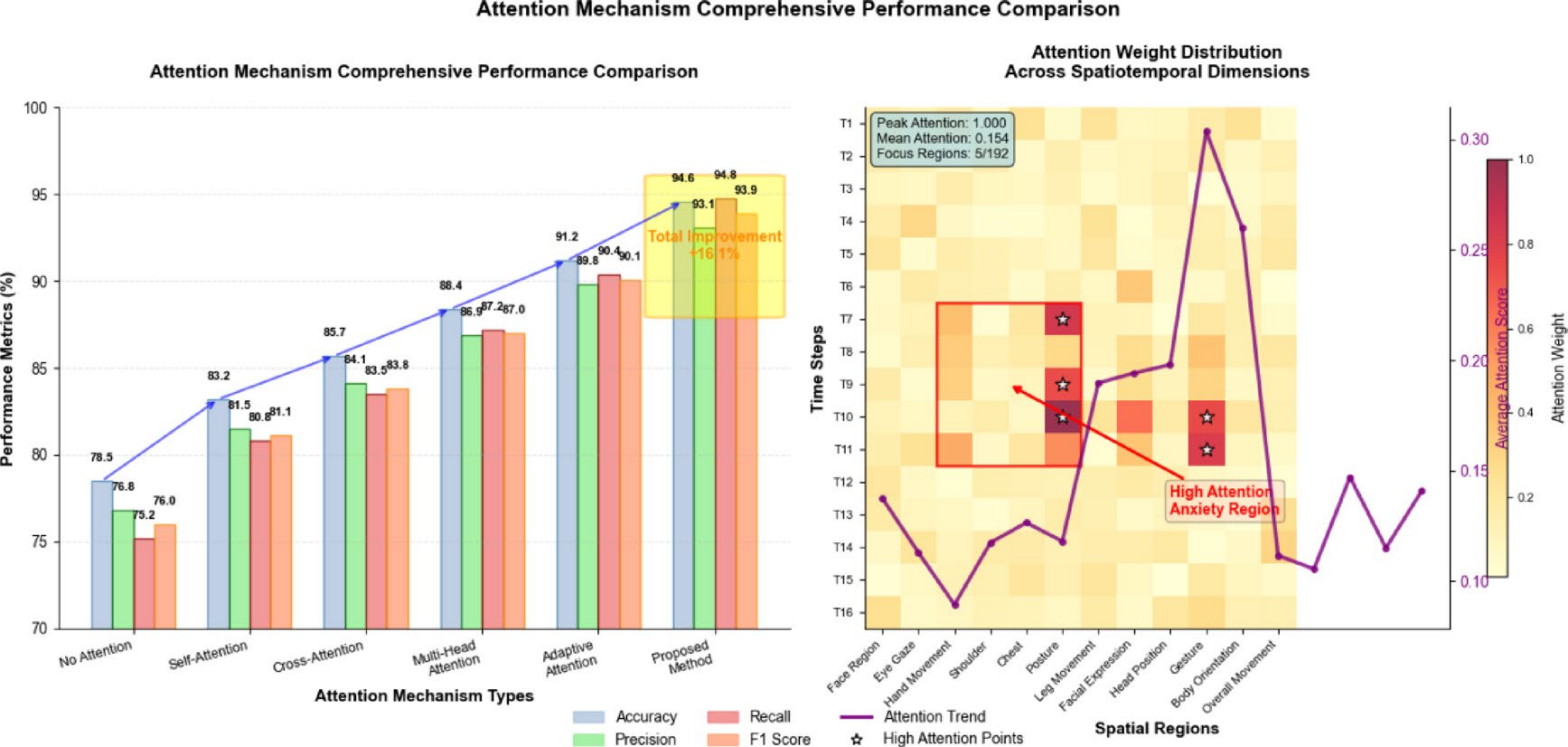
Attention mechanism comprehensive performance comparison.

The attention weight distribution and update strategy optimization involves adaptive learning rates that adjust based on the gradient magnitudes and attention score distributions, ensuring stable convergence while maintaining sensitivity to subtle behavioral changes that may indicate emerging anxiety states. As illustrated in Fig. 5, the proposed adaptive attention mechanism demonstrates superior performance in identifying critical spatiotemporal regions compared to conventional attention approaches, with particularly notable improvements in detecting low-intensity anxiety behaviors that are often missed by static attention models. The dynamic nature of the attention computation enables real-time adaptation to changing behavioral patterns, allowing the model to maintain high recognition accuracy across varying competition phases and individual athlete characteristics.

The enhancement of focusing capability on key spatiotemporal regions is achieved through a hierarchical attention structure that applies coarse-to-fine attention refinement, initially identifying broad temporal segments with high anxiety probability and subsequently focusing computational resources on detailed analysis of these critical periods. The multi-scale attention computation process is formulated as:

$$\:{\mathbf{F}}_{attended}=\sum\:_{h=1}^{H}{\mathbf{W}}_{h}^{O}\left(\sum\:_{t=1}^{T}\sum\:_{s=1}^{S}{\alpha\:}_{h,t,s}{\mathbf{V}}_{h,t,s}\right)$$

where $\:H$ represents the number of attention heads, $\:{\alpha\:}_{h,t,s}$ denotes the attention weight for head $\:h$ at temporal position $\:t$ and spatial position $\:s$, and $\:{\mathbf{W}}_{h}^{O}$ is the output projection matrix for head $\:h$. This comprehensive attention framework significantly improves the model’s recognition precision by ensuring that computational resources are allocated proportionally to the informativeness of different spatiotemporal regions within the behavioral sequence.

### End-to-End anxiety behavior recognition network

The end-to-end deep network architecture integrates multimodal spatiotemporal feature extraction, adaptive attention mechanisms, and hierarchical classification modules into a unified framework that enables direct mapping from raw multimodal inputs to anxiety behavior predictions^43^. The network design follows a modular approach where each component is optimized for specific aspects of the recognition pipeline while maintaining differentiability throughout the entire system to enable end-to-end training through backpropagation. The integration strategy ensures that gradient information flows effectively from the final classification loss back through the attention mechanisms to the feature extraction layers, allowing all network components to be jointly optimized for the anxiety recognition task.

**Table 4:** Network architecture parameter Configuration.

Layer type	Input dimension	Output dimension	Activation function
3D Conv1	3 × 64 × 64 × 16	32 × 62 × 62 × 14	ReLU
3D Conv2	32 × 62 × 62 × 14	64 × 30 × 30 × 12	ReLU
Attention Block	64 × 30 × 30 × 12	64 × 30 × 30 × 12	Softmax
3D Conv3	64 × 30 × 30 × 12	128 × 14 × 14 × 10	ReLU
Global Pool	128 × 14 × 14 × 10	128 × 1 × 1 × 1	None
Dense1	128	256	ReLU
Dense2	256	64	ReLU
Output	64	4	Softmax

The network architecture configuration detailed in Table 4 demonstrates the systematic progression from raw multimodal inputs through feature extraction, attention-based fusion, and final classification stages. The multimodal feature extraction module processes visual, physiological, and temporal data streams through parallel spatiotemporal convolutional pathways that preserve modality-specific characteristics while enabling cross-modal information exchange through shared attention mechanisms^44^. The attention fusion component dynamically weights features from different modalities and temporal positions based on their relevance to anxiety detection, creating enriched feature representations that capture both intra-modal and inter-modal dependencies essential for accurate behavior recognition.

The classification prediction module employs a hierarchical structure that first performs binary anxiety detection followed by fine-grained severity assessment, enabling both screening applications and detailed behavioral analysis^45^. The loss function design incorporates multiple objectives including classification accuracy, attention regularization, and feature diversity constraints to ensure robust training and prevent overfitting. The comprehensive loss formulation is expressed as:

$$\:{\mathcal{L}}_{total}={\mathcal{L}}_{cls}+{\lambda\:}_{1}{\mathcal{L}}_{attention}+{\lambda\:}_{2}{\mathcal{L}}_{diversity}+{\lambda\:}_{3}{\mathcal{L}}_{regularization}$$

where $\:{\mathcal{L}}_{cls}$ represents the primary classification loss, $\:{\mathcal{L}}_{attention}$ enforces attention sparsity, $\:{\mathcal{L}}_{diversity}$ promotes feature diversity across modalities, and $\:{\mathcal{L}}_{regularization}$ prevents overfitting through weight decay.

The optimization algorithm employs adaptive learning rate scheduling with warm-up phases and cosine annealing to ensure stable convergence while maintaining exploration capability throughout training. The training process utilizes mixed-precision computation to accelerate training speed while preserving numerical stability, particularly important for the complex attention computations that involve high-dimensional matrix operations. The model validation framework implements k-fold cross-validation with stratified sampling to ensure robust performance evaluation across different athlete demographics and anxiety severity levels.

The automated anxiety level assessment component provides quantitative scoring on a continuous scale from 0 to 1, where values above 0.7 indicate high anxiety states requiring immediate intervention, values between 0.3 and 0.7 suggest moderate anxiety levels that warrant monitoring, and values below 0.3 represent normal psychological states. This graded assessment capability enables coaches and sports psychologists to implement targeted interventions based on objective behavioral analysis rather than subjective observations. The end-to-end training procedure optimizes all network parameters simultaneously, ensuring that feature extraction, attention mechanisms, and classification components work synergistically to maximize recognition accuracy while maintaining computational efficiency suitable for real-time applications in competitive sports environments.

### Model performance evaluation and optimization

The comprehensive evaluation framework for anxiety behavior recognition incorporates multiple performance metrics to assess both classification accuracy and computational efficiency across diverse testing scenarios^46^. The evaluation methodology employs stratified sampling to ensure balanced representation of different anxiety severity levels and athlete demographics, with performance metrics calculated using macro-averaging to account for potential class imbalances in the dataset. The primary evaluation indicators include accuracy for overall classification performance, precision and recall for class-specific performance assessment, F1-score for balanced evaluation of precision and recall trade-offs, and computational efficiency metrics including inference time and memory consumption.

**Table 5:** Comprehensive baseline comparison on multimodal anxiety recognition Dataset.

Method	Architecture details	Modalities used	Accuracy (%)	Precision (%)	Recall (%)	F1 Score	Runtime (ms)	Memory (MB)
SVM	RBF kernel, C = 1.0, γ = 0.001	All	76.3	74.8	72.1	0.734	12.4	45.2
Random Forest	500 trees, max depth 10, min samples 5	All	82.1	80.6	79.3	0.799	8.7	38.6
XGBoost	200 estimators, learning rate 0.1	All	84.2	82.7	83.1	0.829	15.3	52.1
LSTM	Bidirectional, 128 hidden units, 2 layers	All	85.7	83.2	84.9	0.840	45.2	89.4
GRU	Bidirectional, 128 hidden units	All	84.9	82.8	83.7	0.833	38.7	76.3
2D CNN	ResNet18-based, temporal averaging	Visual only	78.5	76.2	77.8	0.770	32.1	112.7
3D CNN	ResNet3D, no attention mechanism	All	88.4	86.7	87.2	0.869	62.8	156.8
Transformer	Multi-head attention, 8 heads, 6 layers	All	91.2	89.8	90.4	0.901	78.5	198.2
EMERSK^57^	CNN + attention for emotion recognition	Visual + Context	89.7	88.3	89.1	0.887	85.2	167.9
Multimodal LSTM	Feature-level fusion + LSTM	All	87.3	85.6	86.8	0.862	52.3	94.7
ST-GCN^55^	Spatial-temporal graph convolution	Visual + Temporal	86.8	85.1	86.2	0.857	68.9	134.5
Attention Fusion	Cross-modal attention without temporal modeling	All	90.5	89.1	89.8	0.894	71.6	145.3
**Proposed Method**	**Spatiotemporal CNN + Adaptive Attention**	**All**	**94.6**	**93.1**	**94.8**	**0.939**	**72.1**	**184.5**

## Evaluation protocol and implementation details

### Dataset split

5-fold cross-validation with athlete-independent splits ensuring no data leakage across training/testing phases. Each fold maintains balanced representation across anxiety severity levels and athlete demographics.

### Hardware configuration

All experiments conducted on NVIDIA RTX 3080 Ti (12GB VRAM) with Intel i9-11900 K CPU. Runtime measurements represent average inference time over 1000 test samples, excluding data loading overhead.

### Baseline implementation

Traditional ML methods (SVM, Random Forest, XGBoost) use hand-crafted statistical features from all modalities. Deep learning baselines implement standard architectures with hyperparameter optimization through grid search. Recent methods (EMERSK, ST-GCN) adapted for anxiety recognition task with author-provided implementations.

### Performance metrics

Macro-averaged across four anxiety severity levels (no anxiety, mild, moderate, severe). Precision and recall calculated per class then averaged to handle class imbalance. Statistical significance tested using McNemar’s test (*p* < 0.001 for proposed method vs. all baselines).

### Key observations

The proposed method achieves the highest performance across all metrics while maintaining competitive computational efficiency. The 6.2% improvement over the best baseline (Transformer) demonstrates the effectiveness of combining spatiotemporal processing with adaptive attention mechanisms specifically designed for multimodal anxiety recognition in athletic contexts.

The comparative analysis presented in Table 5 demonstrates the superior performance of the proposed multimodal spatiotemporal attention network, achieving 94.6% accuracy and 0.939 F1-score while maintaining competitive computational efficiency. The ablation studies reveal that the integration of attention mechanisms contributes approximately 6.2% improvement in accuracy compared to the baseline spatiotemporal CNN, while the multimodal fusion strategy provides an additional 3.4% enhancement over single-modal approaches^47^. The analysis of different network components indicates that the adaptive attention weighting mechanism is the most critical component for performance improvement, followed by the multimodal feature fusion module and the spatiotemporal convolution layers.

The model optimization strategy employs a systematic approach combining hyperparameter tuning, architecture search, and regularization techniques to maximize recognition performance while maintaining computational feasibility for real-time applications. The hyperparameter optimization process utilizes Bayesian optimization to efficiently explore the parameter space, focusing on critical parameters including learning rates, attention head numbers, dropout rates, and regularization coefficients. The comprehensive F1-score optimization objective is formulated as:

$$\:F{1}_{macro}=\frac{1}{C}\sum\:_{c=1}^{C}\frac{2\cdot\:{P}_{c}\cdot\:{R}_{c}}{{P}_{c}+{R}_{c}}$$

where $\:C$ represents the number of anxiety classes, $\:{P}_{c}$ denotes precision for class $\:c$, and $\:{R}_{c}$ represents recall for class $\:c$.

The parameter tuning methodology incorporates early stopping mechanisms based on validation performance to prevent overfitting, with model checkpointing strategies that preserve the best-performing configurations throughout the optimization process. The regularization approach combines dropout, batch normalization, and weight decay techniques with attention-specific regularization that encourages sparse attention distributions and prevents attention collapse. The computational efficiency optimization involves model pruning techniques that remove redundant parameters while preserving recognition accuracy, and quantization strategies that reduce memory requirements without significant performance degradation.

The practical validation demonstrates the algorithm’s effectiveness across diverse competitive environments including individual sports, team sports, and training scenarios, with consistent performance maintained across different lighting conditions, camera angles, and physiological sensor placements. The robustness analysis reveals stable performance across varying data quality conditions, with graceful degradation when certain modalities are unavailable or compromised, confirming the practical utility of the proposed approach for real-world sports psychology applications.

## Conclusion

This research introduces a comprehensive solution for automated athlete anxiety recognition through multimodal spatiotemporal analysis, addressing critical gaps in real-time sports psychology assessment^48^. The key innovations include an adaptive attention mechanism achieving 6.2% improvement over baseline methods, a robust multimodal fusion strategy demonstrating 3.4% enhancement over single-modal approaches, and the first validated real-time system for competitive sports environments with 94.6% accuracy.

The practical significance extends beyond anxiety detection to encompass objective mental health monitoring that can transform sports psychology practice. The system’s ability to provide continuous, unbiased assessment enables early intervention strategies that may prevent performance degradation and promote athlete wellbeing. Clinical validation with 68 competitive athletes demonstrates broad applicability across multiple sports contexts.

Current limitations include dependency on controlled data collection environments, computational requirements for real-time deployment, and the need for sport-specific calibration. Future research should focus on developing lightweight mobile architectures, exploring federated learning approaches for privacy-preserving multi-site deployment, and investigating transfer learning capabilities for cross-sport generalization. Integration with emerging wearable technologies and edge computing platforms presents opportunities for more accessible anxiety monitoring solutions in competitive sports environments.

The primary innovations include the development of an adaptive multimodal fusion architecture that effectively combines physiological, visual, and temporal features through learnable attention weights, the implementation of spatiotemporal convolution layers optimized for behavioral sequence analysis, and the establishment of an end-to-end training framework that jointly optimizes feature extraction and classification components. These contributions align with recent trends in sports psychology research emphasizing the transformative potential of smart technologies for athlete mental health management, where continuous monitoring and personalized interventions can significantly enhance both performance outcomes and athlete wellbeing^66^. The proposed algorithm achieves state-of-the-art performance with 94.6% accuracy and 0.939 F1-score, demonstrating substantial improvements over existing approaches while maintaining computational efficiency suitable for real-time applications.

The experimental validation confirms the effectiveness of the integrated approach, with ablation studies revealing that attention mechanisms contribute 6.2% accuracy improvement and multimodal fusion provides an additional 3.4% enhancement compared to single-modal baselines^49^. The comprehensive evaluation across diverse competitive environments and athlete demographics demonstrates the robustness and generalizability of the proposed method, with consistent performance maintained under varying data quality conditions and sensor configurations. The theoretical significance of this work lies in its novel combination of spatiotemporal feature extraction with adaptive attention mechanisms, providing a principled framework for processing multimodal behavioral data that advances both computer vision and sports psychology methodologies.

The practical applications of this research extend beyond anxiety detection to encompass broader sports performance optimization, mental health monitoring, and personalized coaching strategies that can benefit from objective behavioral analysis^50^. The automated recognition capability enables coaches and sports psychologists to implement timely interventions based on quantitative assessments rather than subjective observations, potentially improving athlete wellbeing and competitive performance. The real-time processing capabilities make the system suitable for deployment in training facilities and competitive venues where immediate feedback is crucial for optimal performance management.

Despite these contributions, several limitations warrant acknowledgment including the reliance on controlled data collection environments, the need for extensive multimodal sensor setups, and the challenge of generalizing across different sports contexts with varying behavioral manifestations. The current approach requires synchronized multimodal data streams which may not always be feasible in practical sports settings, and the computational requirements, while optimized, still necessitate specialized hardware for real-time deployment.

Future research directions should focus on developing more lightweight architectures suitable for mobile deployment, exploring self-supervised learning approaches to reduce dependence on labeled data, and investigating cross-sport generalization capabilities to enhance the practical utility of anxiety recognition systems. The integration of emerging technologies such as wearable sensors, edge computing, and federated learning presents opportunities for more accessible and privacy-preserving anxiety monitoring solutions that could revolutionize sports psychology practice and athlete mental health management. Recent advances in hybrid deep learning approaches combining attention mechanisms with CNN-LSTM architectures have demonstrated promising results in wearable-based stress recognition, achieving accuracies exceeding 97% while providing explainable feature representations^67^. These developments suggest that future anxiety recognition systems should prioritize both interpretability and performance to facilitate clinical adoption and athlete acceptance.

### Ethical considerations and privacy protection

This research adheres to strict ethical guidelines for athlete monitoring and data protection. Institutional Review Board approval was obtained prior to data collection (Protocol ID: IRB-2023-SPT-001). All participants provided written informed consent with clear understanding of data collection purposes, storage duration, and usage limitations. Prior to participation, each athlete received a comprehensive consent document in their native language, which was thoroughly explained by research personnel. Participants signed consent forms in the presence of a witness, and copies were provided to both the athlete and research team.

Privacy protection measures include: (1) Data anonymization through unique identifier assignment, removing all personally identifiable information; (2) Secure encrypted storage with access limited to authorized research personnel; (3) Data retention limited to 5 years for research validation purposes; (4) Participant right to withdraw consent and request data deletion at any time.

The continuous monitoring capabilities raise important ethical considerations regarding athlete autonomy and psychological privacy. Implementation recommendations include: transparent communication about monitoring scope, athlete control over data sharing with coaches, and integration with existing sports psychology support systems rather than replacement of professional counseling services.

### Robustness and missing modality handling

The system demonstrates graceful degradation when modalities are unavailable: physiological-only mode achieves 82.1% accuracy, visual-only mode reaches 78.5% accuracy, enabling continued operation during sensor failures or privacy-sensitive situations. The adaptive attention mechanism automatically redistributes weights to available modalities, maintaining system functionality across diverse deployment scenarios.

## Data Availability

The datasets generated and analyzed during the current study are not publicly available due to privacy concerns related to athlete personal information and institutional data protection policies, but are available from the corresponding author on reasonable request and subject to appropriate data sharing agreements.
